# Macrophage Migration Inhibitory Factor—An Innovative Indicator for Free Flap Ischemia after Microsurgical Reconstruction

**DOI:** 10.3390/healthcare9060616

**Published:** 2021-05-21

**Authors:** Ioannis-Fivos Megas, David Simons, Bong-Sung Kim, Christian Stoppe, Andrzej Piatkowski, Panagiotis Fikatas, Paul Christian Fuchs, Jacqueline Bastiaanse, Norbert Pallua, Jürgen Bernhagen, Gerrit Grieb

**Affiliations:** 1 Department of Plastic Surgery and Hand Surgery, Gemeinschaftskrankenhaus Havelhoehe, Kladower Damm 221, 14089 Berlin, Germany; fivos.megas@gmail.com (I.-F.M.); jacqueline.bastiaanse@havelhoehe.de (J.B.); 2 Burn Center, Department of Plastic Surgery and Hand Surgery, Medical Faculty, RWTH Aachen University, Pauwelsstrasse 30, 52074 Aachen, Germany; simonsd@gmx.de (D.S.); bong-sung.kim@usz.ch (B.-S.K.); piatkowski.de.grzymala@gmail.com (A.P.); npallua@ukaachen.de (N.P.); 3 Institute of Biochemistry and Molecular Cell Biology, Medical Faculty, RWTH Aachen University, Pauwelsstrasse 30, 52074 Aachen, Germany; juergen.bernhagen@med.uni-muenchen.de; 4 Department of Plastic Surgery and Hand Surgery, University Hospital Zurich, Rämistrasse 100, 8091 Zurich, Switzerland; 5 Department of Anesthesiology, Intensive Care Medicine and Pain Therapy, University Hospital Würzburg, Josef-Schneider-Straße 2, 97080 Würzburg, Germany; christian.stoppe@gmail.com; 6 Maastricht University Medical Center, Department of Plastic and Reconstructive Surgery, 6229 HX Maastricht, The Netherlands; 7 Department of Surgery, Campus Charité Mitte and Campus Virchow-Klinikum, Charité—Universitätsmedizin, Corporate Member of Freie Universität Berlin, Humboldt-Universität zu Berlin and Berlin Institute of Health, Augustenburger Platz 1, 13353 Berlin, Germany; panagiotis.fikatas@charite.de; 8 Burn Center, Department of Plastic Surgery and Hand Surgery, University of Witten/Herdecke, Kliniken der Stadt Köln, Ostmerheimer Str. 200, 51109 Köln, Germany; fuchsp@kliniken-koeln.de; 9 Vascular Biology, Institute for Stroke and Dementia Research (ISD), LMU University Hospital, Ludwig-Maximillian-University (LMU), Feodor-Lynen-Straße 17, 81377 Munich, Germany; 10 German Center for Cardiovascular Diseases (DZHK), Partner Site Munich Heart Alliance, 80336 Munich, Germany; 11 SyNergy Excellence Cluster, 81377 Munich, Germany

**Keywords:** macrophage migration inhibitory factor (MIF), free flap surgery, innovative surgical methods, microanastomosis, ischemia

## Abstract

(1) Background: Nowadays, the use of microsurgical free flaps is a standard operative procedure in reconstructive surgery. Still, thrombosis of the microanastomosis is one of the most fatal postoperative complications. Clinical evaluation, different technical devices and laboratory markers are used to monitor critical flap perfusion. Macrophage migration inhibitory factor (MIF), a structurally unique cytokine with chemokine-like characteristics, could play a role in predicting vascular problems and the failure of flap perfusion. (2) Methods: In this prospective observational study, 26 subjects that underwent microsurgical reconstruction were observed. Besides clinical data, the number of blood leukocytes, CRP and MIF were monitored. (3) Results: Blood levels of MIF, C-reactive protein (CRP) and leukocytes increased directly after surgery. Subjects that needed surgical revision due to thrombosis of the microanastomosis showed significantly higher blood levels of MIF than subjects without revision. (4) Conclusion: We conclude that MIF is a potential and innovative indicator for thrombosis of the microanastomosis after free flap surgery. Since it is easy to obtain diagnostically, MIF could be an additional tool to monitor flap perfusion besides clinical and technical assessments.

## 1. Introduction

Reconstruction using free microsurgical flaps is a standard operative procedure to close and reconstruct various skin and soft tissue defects. Still, thrombosis of the microanastomosis is one of the most fatal postoperative complications, requiring immediate operative revision for flap salvage. Although different technical devices are used to monitor postoperative flap perfusion, clinical evaluation of the flap seems to remains the gold standard [1]. However, circulating blood biomarkers might be a promising additional tool for this purpose, since they are rapidly released upon stimulus and easy to obtain.

The protein mediator macrophage migration inhibitory factor (MIF) might be a novel tool in this context. MIF is a structurally unique pleiotropic cytokine, involved in acute and chronic inflammatory processes and cancer [2,3,4]. Over the last few decades, it has become clear that MIF functions as a chemokine-like cytokine, which promotes the directed migration and recruitment of leukocytes to inflammatory sites [5,6]. MIF interacts with surface CD74, but the leukocyte recruitment is mediated by a non-cognate binding to the chemokine receptors CXCR2 and CXCR4 [5], leading to vascular diseases such as atherosclerosis [6,7]. Furthermore, MIF has been demonstrated to be involved in endothelial progenitor cell mobilisation after free flap surgery [8] and seems to be involved in flap vascularization in a murine model [9]. In addition, MIF has proven to serve as a promising potential biomarker in different diseases throughout different clinical fields [10,11]. Due to its affinity to the vascular system, MIF may play a role for indicating flap ischemia and thrombosis.

In this study, we focus on MIF as a potential and beneficial tool to evaluate and predict critical flap perfusion. Higher MIF values were found in the postoperative course of subjects needing surgical revision, which confirmed the potential of MIF as biomarker in free flap surgery.

## 2. Materials and Methods

### 2.1. Subjects

This prospective observational study included 26 subjects who underwent free flap surgery at the Department of Plastic Surgery and Hand Surgery, Burn Center of the RWTH Aachen University (Table 1). Ten of the subjects were female. The mean age of all subjects was 47.8 years, ranging from 19 to 71. Five subjects needed a surgical flap revision of the microanastomosis. Two flaps were lost due to necrosis. In one case, microsurgical problems already occurred during the initial operation.

Exclusion criteria were: age <18 years, multiple flap surgeries, any form of hematological disease, smoking and acute illness or infection.

None of the patients had taken non-steroidal anti-inflammatory drugs, oral anticoagulation or platelet aggregation inhibitors. Patients received anti-thrombosis prophylaxes using 5000 UI of low-molecular-weight heparin subcutaneously on a daily basis.

Blood samples were collected after written informed consent of the concerning patients and with the approval of the local ethics committee.

### 2.2. Methods

Blood samples were taken by venous puncture 24 h prior to the operation and 3, 6, 12, 24, 36, 48, 72, 96 and 120 h after the operation for MIF determination. Leukocyte and C-reactive protein (CRP) levels, being very easy to obtain, were monitored following a clinical standard procedure 24 h prior to the operation and 24, 48 and 120 h after the operation.

Blood samples (collected in citrate tubes) were spun at 3500× *g* for 10 min. Plasma was stored at −80 °C until analysis. MIF concentrations were determined as published previously [12,13]. Leukocyte and CRP levels were routinely determined by the clinical chemistry laboratory of the University Hospital of the RWTH Aachen, Germany. Blood analyses were not performed blinded.

### 2.3. Statistics

Data are presented in mean ± standard deviation (SD). For statistical analysis, significance was evaluated using a 1-way-analysis of variance (ANOVA) in the case of more than two groups and the Student’s *t*-test (two groups); *p*-values < 0.05 were considered significant.

## 3. Results

In this study, we investigated whether MIF is a potential indicator for postoperative flap ischemia after free flap surgery.

**Figure 1:**
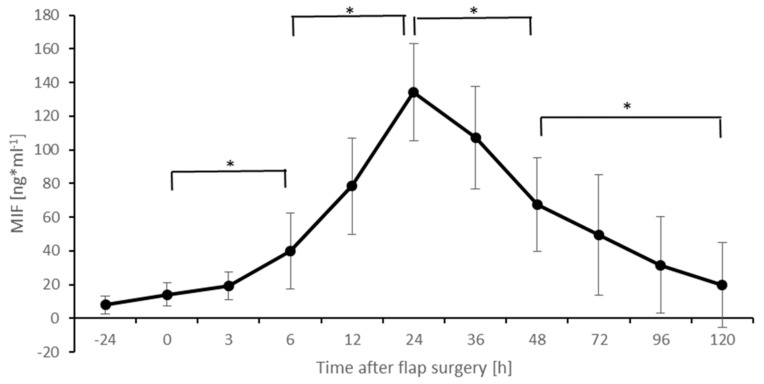
Postoperative MIF levels. Columns indicate mean values; error bars refer to the corresponding standard deviations (* *p* ≤ 0.05).

The perioperative levels of MIF are shown in Figure 1. Six hours after surgery, MIF levels show a significant increase (*p* < 0.05), that peak at 24 h postoperatively (*p* < 0.05). This is followed by a significant decrease in MIF levels after 48 h (*p* < 0.05) and 120 h (*p* < 0.05) after surgery. The location of the defect or flap did not have a significant effect on the MIF levels (data not shown).

**Figure 2:**
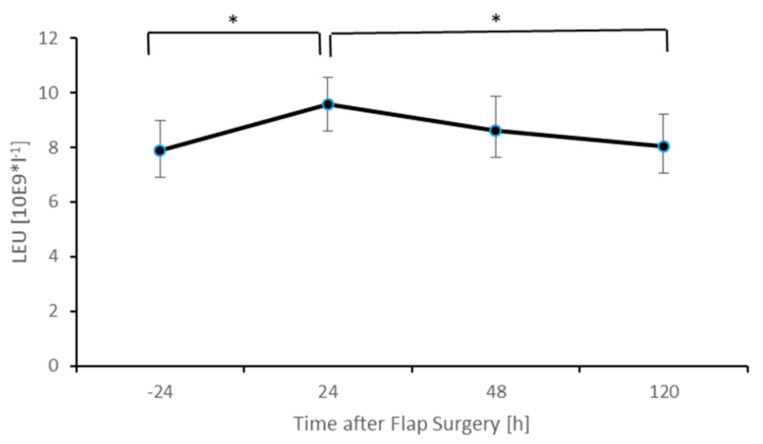
Postoperative levels of leukocytes. Columns indicate mean values; error bars refer to the corresponding standard deviations (* *p* ≤ 0.05).

Figure 2 demonstrates the level of leukocytes pre- and postoperatively. A slight but significant (*p* < 0.05) increase in leukocytes can be observed 24 h after surgery, followed by a delayed but significant decrease (*p* < 0.05) at 120 h after the free flap surgery. After 120 h, levels of leukocytes almost fall back to baseline values (7.9 vs. 8.1 LEU [10^9^*l−1]; *p* = 0.61).

**Figure 3:**
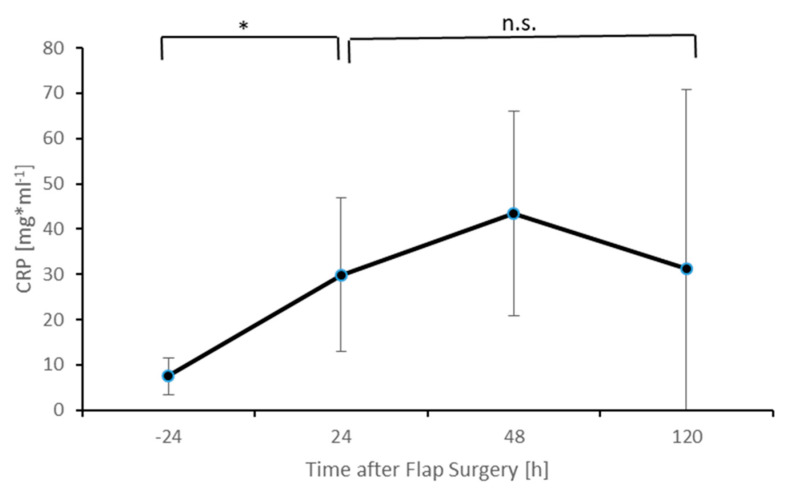
Postoperative CRP levels. Columns indicate mean values; error bars refer to the corresponding standard deviations (* *p* ≤ 0.05; n.s. = not significant).

The levels of CRP are shown in Figure 3. As MIF and leukocyte levels, CRP levels show a significant increase (*p* < 0.05) 24 h after surgery. Further postoperative monitoring showed no significant change of CRP levels.

**Figure 4:**
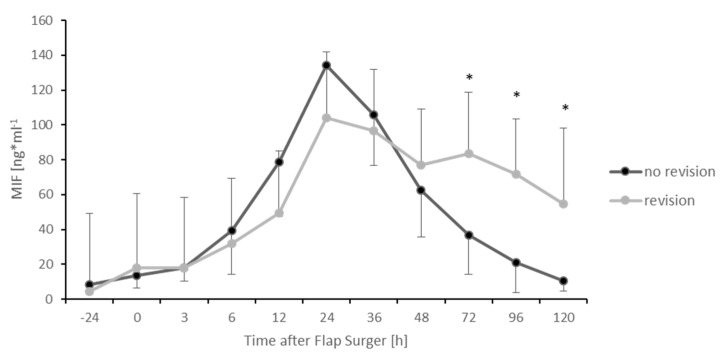
Postoperative MIF levels divided into two groups. Subjects that needed surgical revision due to thrombosis of the microanastomosis (grey) and subjects without surgical revision (black). Error bars refer to the corresponding standard deviations (* *p* ≤ 0.05).

To determine the role of MIF during and after necessary surgical revision, we divided the group into subjects that needed surgical revision due to thrombosis of the microanastomosis and subjects without surgical revision. With respect to MIF levels, the data are shown in Figure 4.

**Figure 5:**
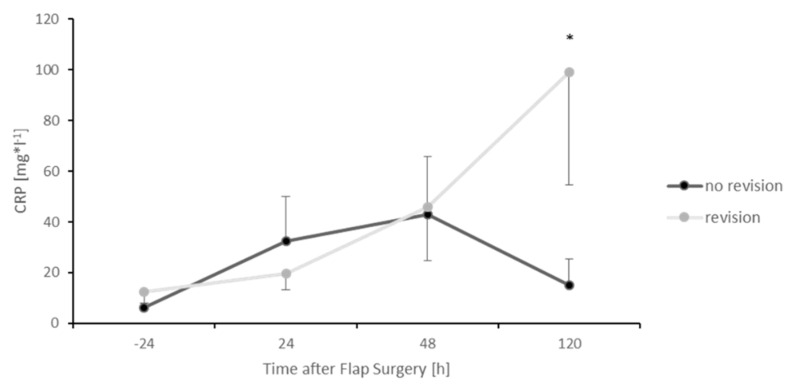
Postoperative CRP levels divided into two groups. Subjects that needed surgical revision due to thrombosis of the microanastomosis (grey) and subjects without surgical revision (black). Error bars refer to the corresponding standard deviations (* *p* ≤ 0.05).

**Figure 6:**
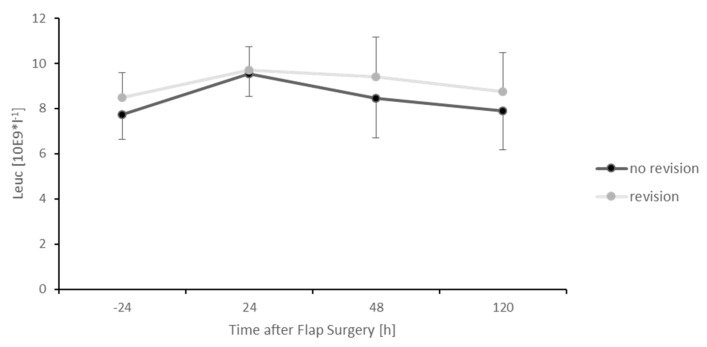
Postoperative levels of leukocytes divided into two groups. Subjects that needed surgical revision due to thrombosis of the microanastomosis (grey) and subjects without surgical revision (black). Error bars refer to the corresponding standard deviations (* *p* ≤ 0.05).

The group needing revision surgery demonstrated significantly higher MIF values during the postoperative course, as demonstrated at 72 (*p* < 0.05), 96 (*p* < 0.05) and 120 (*p* < 0.05) hours after the initial free flap surgery compared to the subjects without surgical revision. This also applied to CRP levels 120 h after surgery (*p* < 0.05). In addition, no significant difference of MIF levels could be detected between an arterial or venous thrombosis of the microanastomosis (data not shown). MIF levels of all subjects are additionally presented in Figure S1. The remaining CRP levels and all Leukocyte levels in contrast did not show any significant difference between those two groups (Figure 5 and Figure 6).

## 4. Discussion

This study for the first time addressed the potential use of the cytokine MIF as a potential marker for flap ischemia following free flap surgery.

Free flap surgery is a routinely used method to cover various defects. If a problem of the microanastomosis occurs, immediate surgical intervention is essential for flap salvage [14]. Different techniques have been described to monitor postoperative flap perfusion such as the handheld Doppler ultrasound probe, laser Doppler flowmetry, nuclear medicine, near-infrared spectrometry, perfusion photoplethysmography, surface temperature measurement, confocal microscopy, white light spectroscopy, subcutaneous pH measurement, multispectral spatial frequency domain imaging, orthogonal polarized light, sidestream dark field imaging, CO2 monitoring, pulse oximetry, fluorometry, injectable biosensors and Cook–Swartz Doppler [14,15,16,17,18,19,20,21,22,23,24,25,26,27,28]. However, clinical evaluation of the flap seems to remain the gold standard [1].

In addition to technical devices, the use of biomarkers is of increasing interest as they may further increase the sensitivity and specificity in the clinical decision-making process. Koerdt et al. monitored serum levels of procalcitonin in head and neck free flaps [29]. A higher plasma level of IL-8 and TNF-α after venous thrombosis in a canine model were observed by Du et al. [30]. Hill et al. described low hemoglobin and hematocrit levels as preoperative risk markers before free flap surgery [31]. Another clinical study by Kloeters et al. showed that higher levels of prothrombin fragment and thrombin–antithrombin III complex due to delayed microsurgical reconstruction present a higher risk of flap failure [32].

Generally, in this study, an increase in MIF levels can be observed 24 h after the operation of free flaps. This also applies to the levels of leukocytes and CRP, indicating a more general inflammation due to the extended operation. However, this is well in line with observations our group observed before [8].

Furthermore, MIF already has been described to be released after hypoxia or ischemia and to be involved in ischemia/reperfusion injury [33].

However, when comparing the group of subjects in this study needing surgical revision with the group of subjects without revision of the microanastomosis, higher MIF values were detected 72, 96 and 120 h after the primary operation for the group that needed revision. Thus, MIF could be interpreted as a potential indicator for the need of surgical revision, since all surgical revised subjects demonstrated problems of the microanastomosis. In addition, this effect was also seen with respect to CRP levels after 120 h. However, the conclusion of this effect is limited for CRP due to the limited time points in this study. This is in line with the observation of Wright et al. who interpreted a CRP peak four days after flap surgery as an indicator for infection and need for further surgery [34]. However, CRP represents a rather general marker for inflammation. Since MIF seems to act very specifically after ischemia/reperfusion [33], its role as a biomarker in this context could be very prominent. Nevertheless, in all surgically revised cases, the clinical decision for revision was made before a second rise of MIF-levels could be observed. In this experimental setting with only a moderate number of subjects, blood samples were stored until analysis with an ELISA to determine the amount of MIF. For a “true” clinical setting with potential decisions concerning revision surgery, this test procedure would be too slow. A quick “MIF” bedside test could be the potential solution for this time problem. However, the development of such a test is connected with high costs and would only be of interest if the sensitivity and specificity of the test are high enough. Thus, the role of the cytokine MIF as a biomarker in free flap surgery needs to be elucidated further.

## 5. Conclusions

We conclude that the cytokine MIF is a potential and innovative marker for flap ischemia due to thrombosis of the microanastomosis and thus, a potential indicator for necessary surgical revision after free flap surgery. Since MIF-levels are easy to obtain, we are convinced that it could play a role as an additional tool to monitor flap perfusion besides clinical and technical assessments. However, besides faster high quality test procedures, more clinical studies with larger cohorts are needed to underpin the role of MIF in this context.

## Figures and Tables

**Table 1:** Demographical and clinical characteristics of patient cohort.

Patients	Age	Sex	Region of Skin/Soft Tissue Defect	Free Flap Procedure	Revision Necessary	Total Flap Loss
		(M/F)			(y/n)	(y/n)
Patient 1	66	M	elbow	antero lateral thigh flap	n	n
Patient 2	41	M	hand	antero lateral thigh flap	n	n
Patient 3	53	M	abdomen	latissimus dorsi flap	n	n
Patient 4	42	F	lower leg	radial forearm flap	n	n
Patient 5	55	M	foot	latissimus dorsi flap	n	n
Patient 6	19	M	hand	parascapular	n	n
Patient 7	50	M	lower leg	latissimus dorsi flap	n	n
Patient 8	25	M	face	gracilis flap	n	n
Patient 9	27	M	lower leg	latissimus dorsi flap	n	n
Patient 10	50	M	lower leg	antero lateral thigh flap	n	n
Patient 11	53	M	lower leg	gracilis flap	n	n
Patient 12	46	F	breast	diep flap	n	n
Patient 13	71	F	lower leg	radial forearm flap	n	n
Patient 14	59	M	lower leg	antero lateral thigh flap	y	n
Patient 15	66	M	lower leg	gracilis flap	y	n
Patient 16	63	F	breast	diep flap	n	n
Patient 17	71	F	lower leg	gracilis flap	n	n
Patient 18	51	F	breast	diep flap	n	n
Patient 19	59	M	foot	antero lateral thigh flap	n	n
Patient 20	48	F	breast	diep flap	n	n
Patient 21	29	M	knee	gracilis flap	y	y
Patient 22	52	F	breast	diep flap	n	n
Patient 23	26	M	axilla	antero lateral thigh flap	n	n
Patient 24	30	M	lower leg	antero lateral thigh flap	y	y
Patient 25	42	F	breast	diep flap	y	n
Patient 26	51	F	breast	diep flap	n	n
	Age					
Mean	47.885					
Range	19 to 71					
Median	50.5					
SD	14.72					

## Data Availability

Not applicable.

## Supplementary Materials

The following are available online at https://www.mdpi.com/article/10.3390/healthcare9060616/s1, Figure S1: MIF-levels of all subjects. Levels of subjects needing surgical revision are connected with lines.
