# Supplementary figures and images for: Macrophage Migration Inhibitory Factor—An Innovative Indicator for Free Flap Ischemia after Microsurgical Reconstruction
Source: Healthcare (Basel). 2021 May 21;9(6):616. doi: 10.3390/healthcare9060616 (PMC8223971; doi:10.3390/healthcare9060616)

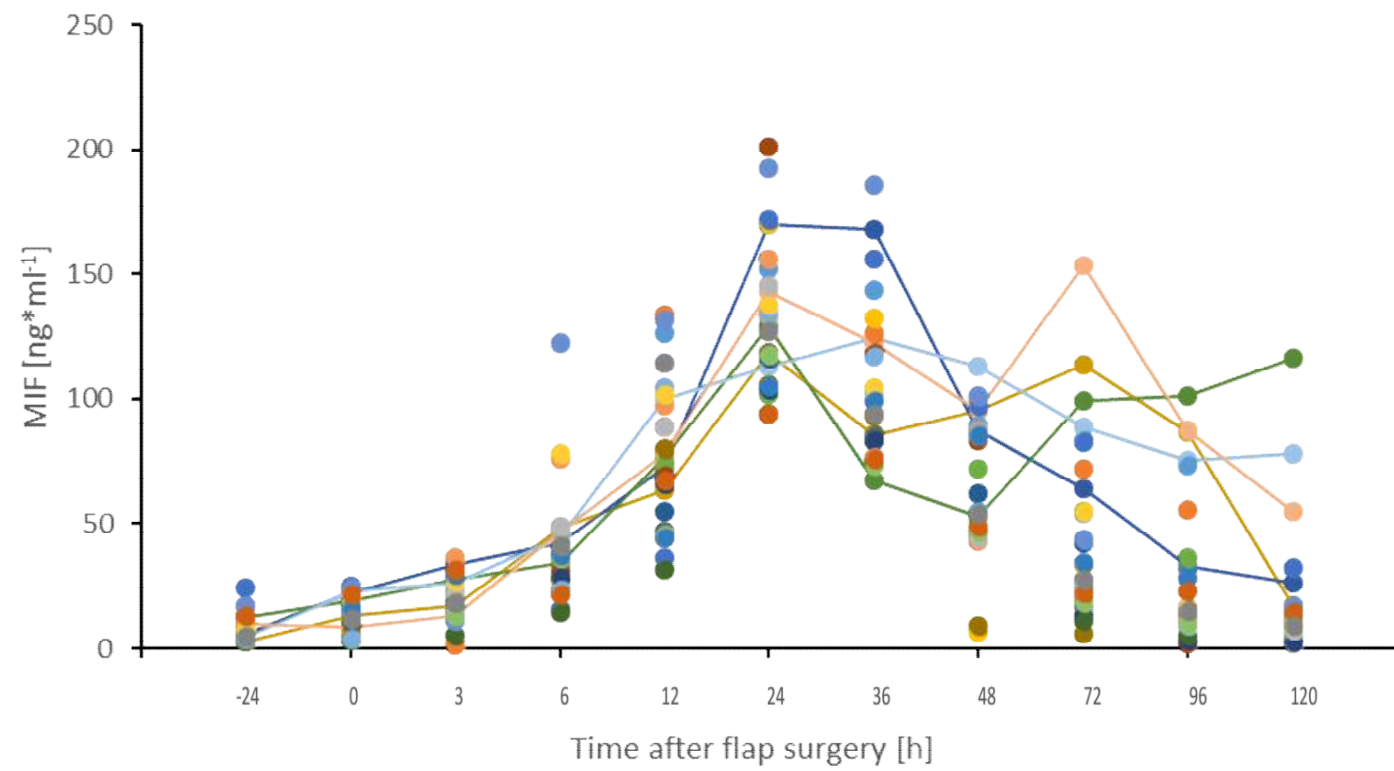

Supplement: Supplementary file 1 [file healthcare-09-00616-s001.zip › healthcare-1214020-supplementary.pdf]
